# Capturing the fungal diversity in manure, lagoons, troughs, and flies at a commercial dairy

**DOI:** 10.3389/fmicb.2026.1794875

**Published:** 2026-04-13

**Authors:** Tawni L. Crippen, Dongmin Kim, Sonja L. Swiger, Robin C. Anderson, Ryan J. Arsenault

**Affiliations:** 1 Southern Plains Agricultural Research Center, Agricultural Research Service, U.S. Department of Agriculture, College Station, TX, United States; 2 Department of Entomology, Texas A & M University, College Station, TX, United States; 3 Entomology Extension, Texas AgriLife, Texas A&M University, College Station, TX, United States

**Keywords:** bovine, Diptera, effluent ponds, microbiome, pathogens

## Abstract

The microbiomes within dairy facilities that could serve as reservoirs for beneficial and pathogenic fungi have not been extensively explored. Though fungi can cause food safety and animal health issues, they also represent species contributing to bovine digestion and environmental nutrient cycling. This study investigated whether fungal communities from specific elements at a working dairy differed between cross-vent or flow-through, free stall barn management systems and defined the possible pathogen locations. Shotgun metagenomics was carried out on manure, lagoons, troughs, and fly samples from the barns. The diversity of species was not significantly affected by management systems, except between lagoon communities. Flies carried the highest number of unique fungal species and the most abundant potential mammalian pathogens, but there was a lack of overlapping pathogen profiles between flies and the other dairy components. Thus, it remains unclear whether the species are being efficiently exchanged between these different components of the dairy environment, mechanically or biologically. Manure harbored the most opportunistic pathogenic species, lagoons harbored the most plant pathogens and beneficial species, and troughs had the most innocuous or understudied species. The results allow dairy managers to consider advantageous management systems and focus on fungal mitigation efforts at appropriate locations within the dairy.

## 1 Introduction

The global demand for meat and dairy products continues to rise with growing human populations (Turk, 2016). The US Department of Agriculture, National Agricultural Statistics Service reported in 2022 that there were almost 28,000 licensed dairy herds in the United States, consisting of over 9,400,000 heads of dairy cattle (ERS, 2023). Microorganisms in dairy products can present public health and food safety issues. Pasteurization can alleviate concerns in milk products, but some spore-forming fungi may survive and still require constant surveillance to maintain the health of the cattle and the dairy facility environment (Oliver et al., 2005). Pathogens can be introduced by ingestion of contaminated feed and subsequent amplification in bovine hosts and dissemination in the farm environment through water and waste systems. The mitigation of this contamination could be improved by interrupting the inadvertently maintained reservoirs of foodborne pathogens. But first they must be identified. Some past studies have been conducted to determine where the associated reservoirs of foodborne pathogens are found in dairies (Oliver et al., 2005). However, next-generation sequencing provides a broader and more in-depth identification of dairy fungal communities.

Dairy cattle living in a free stall type facility are not restrained and may move around the enclosure and interact with other cattle. The conventional management arrangement is an outdoor enclosure allowing natural air flow-through (FT) in the barn, possibly assisted by fans in hot climate areas. Cross-vent (CV) barns, however, are a more structured management arrangement. Airflow is controlled with baffles that hang from the ceilings to redirect air at a greater velocity into the stalls. Also, there are exhaust fans on one wall and air-intake by evaporative panels on the opposing wall. The purpose is to regulate humidity and temperature for the benefit of the dairy cattle to alleviate stress and to assure good, consistent milk production (Wolf and McBride, 2018). In a CV barn, the air flows parallel to the free stalls and in between cows, cooling them when lying down (Wolf and McBride, 2018). The CV management system not only reduces heat in the barn but can also decrease the indoor concentration of gases. Within such agricultural settings, microbes can be exchanged between various elements and surfaces of the barns (Bradford et al., 2013). Fungi are often overlooked in these anthropogenic systems. In these environments, fungi must contend with common man-made physical and chemical disruptions while conducting their essential ecological functions of energy and nutrient recycling (Newbound et al., 2010). We wondered whether the environmental differences dictated by the dairy management systems (CV versus conventional FT) of the barns could influence the fungal biome structure. In particular, the distribution of fungi that cause food safety and animal health concerns.

While fungal communities in dairy products (e.g., milk, butter, and cheese) have been examined, those in environmental reservoirs within dairy facilities remain poorly characterized (Garnier et al., 2017; Fatima, 2021; Ferrocino et al., 2022). Here, we investigated the presence of fungal species associated with common elements with which cattle interacted, as well as their association with the free stall management systems conducted at the facilities. Further, since insects harbor numerous and diverse microbiota, which are heavily influenced by their habitats, the house flies (*Musca domestica* L.) and stable flies (*Stomoxys calcitrans* L.) (Diptera: Muscidae) were also sampled. The two species of flies examined represent different feeding behaviors. The house fly is a non-biting fly adapted to feeding on a wide range of food sources by expectorating salivary digestive enzymes to ensure that the food source is liquified for sponging. Whereas the stable fly is a hematophagous fly that is adapted for piercing the skin and feeding on blood. The purpose of this study was to: (1) investigate the occurrence of fungal species within specific environmental elements at a working dairy implementing two barn management systems, (2) determine where possible pathogenic fungi resided, and (3) discern the overlap of species between the different elements and thus likely pathways of spread of pathogenic species.

## 2 Materials and methods

### 2.1 Site and dairy health records

The dairy was in North Texas, where in the summer (June–August) of 2018, it was dry, and rainfall was erratic, with a mean of 0.012 cm precipitation during that summer. Temperatures were high with a mean day and night temperature of 34.2 and 28.5 °C, respectively.^1^ The sandy to loamy soils in the area supported a range of prairie grasses. This was a large dairy (≥500 head) that maintained Holstein cattle that were milked twice a day. The heifers were pastured or dry-lotted, dry or infirm cows were in free-stall FT barns, and milk cows were in a side-by-side open-stalled (1.2 × 2.8 m) CV barn. Cattle were fed forage-based total mixed rations comprising corn silage, sorghum, and coastal grass twice daily. There was year-round automated lighting (LED lights 18:6 h L:D) in the CV barn, which also contained a feedline water soaker system with baffle curtains to conduct airflow directly over the cows. Both the CV and FT barns were slightly sloping and manually cleaned by vacuum. The CV wash system was integrated with the milking parlor wash and discharged into multiple waterways that emptied into three sequential, uncovered anaerobic/facultative lagoons that were naturally aerated. Manure waste for the FT barn was manually collected and trucked to a separate sequential three-lagoon system connected by waterways.

Health records for this dairy are presented as the percentage of the herd affected during the month prior, the month of, and the month after the samples were collected from the dairy, respectively. Records showed that 0.31, 0.29, and 0.64% cattle died; 6.31, 7.00, and 7.41% had mastitis; 0.36, 0.36, and 0.33% had metritis; in 1.00, 0.93, and 0.93%, abortions had occurred; 0.34, 0.52, and 0.72% retained placentas; 0.52, 0.38, and 0.47% presented with pneumonia; 0.24, 0.17, and 0.38% had ketosis; 0.17, 0.03, and 0% were bloated; 0.12, 0.17, and 0.19% displayed abomasal displacement; 0.02, 0.03, and 0.02% had hemorrhagic bowel disease; 0.07, 0, and 0.03% showed milk fever; 0.10, 0.02, and 0.19% had fever (no reported cause); 0, 0, and 0.02% had pinkeye; and 10.40, 9.76, and 9.95% exhibited lameness.

### 2.2 Sampling design

To simplify multiple samples taken from CV manure, CV lagoon, CV trough, CV house fly, CV stable fly, FT manure, FT lagoon, FT trough, FT house fly, and FT stable fly, they are referred to as the elements within the dairy. The combined results from each of the two barn systems of the manure, lagoon, trough, house fly, and stable fly are referred to as the components within the dairy. Finally, the combined results of all the elements from one of the two barn systems, CV or FT, are referred to as the management system. Samples were collected from the elements of the two management system areas. All samples taken were aseptically collected into 100 mL sterile specimen cups in June 2018. One hundred aliquots of ~10 g manure were collected from stalls and alleyways in the CV and 100 from the FT barns. A mixture of 20 particulate and water (100 mL) samples was aseptically collected from the shoreline perimeter along the water–soil interface of the FT lagoon, and another 20 were collected from the CV lagoon systems. An aliquot (500 mL) of water was sampled from 10 different troughs per management system location CV (*n* = 10) and FT (*n* = 10). Arthropods were gathered using a sweep net through the alleyways beside the stalls. These samples were aseptically sorted by species at the laboratory. Only house flies and stable flies were retained for analysis. Each species was combined into groups of 10 flies per sample. A total of five CV house fly, five FT house fly, five CV stable fly, and three FT stable fly samples were prepared for sequencing analyses.

### 2.3 DNA extraction, sequencing, and bioinformatics analyses

DNA was extracted from manure or lagoon samples using the MP FastDNA Spin Kit for Feces (MP Biomedicals, Irvine, CA), according to the manufacturer’s instructions, and standardized to 50 ng/μL prior to combining into composite samples. Composite samples were made, consisting of groups of 5 samples of manure, resulting in 20 CV manure (*n* = 20) and 20 FT manure (*n* = 20) samples, in total. The trough water samples were vacuum-filtered via sterile bottle top 0.22-μm filters (Corning Inc. Glendale, AZ), from which the filters were retained for DNA extraction. Organic phenol–chloroform methodology was used for DNA extraction from both the flies and the trough filters. This involved cell lysis in Carlson lysis buffer (Fisher Scientific, Fair Lawn, NJ) then disruption using a 0.1-mm zirconium beads (OPS diagnostics, Lebanon, NJ) at 6 m/s for 40 s on a FastPrep-24 homogenizer (MP Biomedicals, Irvine, CA), followed by incubation for 1 h at 37 °C with 15 mg/mL lysozyme (Fisher Scientific, Fair Lawn, NJ). Then incubation was performed for 16 h at 65 °C with 10% sodium dodecyl sulfate (Fisher Scientific, Fair Lawn, NJ) and 100 μg/mL proteinase K (Fisher Scientific, Fair Lawn, NJ). The top layer was retained after centrifugation for 1 min at 17,000 x g. An equal volume of 25:24:1 phenol/chloroform/isoamyl alcohol (Sigma-Aldrich, St. Louis, MO) was added and centrifuged 6 min at 17,000 x g at 4 °C. The aqueous layer was retained, and 100 μg/mL ribonuclease A (Amresco Inc., Solon, OH) was added and incubated for 30 min at 37 °C. An equal volume 24:1 chloroform/isoamyl alcohol (Sigma-Aldrich, St. Louis, MO) was added and centrifuged for 6 min at 17,000 x g at 4 °C, and the aqueous layer was retained. This step was repeated. The DNA was precipitated with isopropyl alcohol and centrifuged for 25 min at 17000 x g at 4 °C. The pellet was washed with 70% ethanol, followed by centrifugation for 25 min at 17000 x g at 4 °C.

Extracted DNA from all samples (*n* = 118) was normalized to 50 ng/μL before sequencing using a microvolume spectrophotometer (DeNovix Inc., Wilmington, DE) and then stored at −20 °C until analyses. Sequencing was performed using whole-genome shotgun metagenomics utilizing CosmosID services (CosmosID Inc., Germantown, MD) with run controls of *Allobacillus halotolerans* and *Imtechella halotolerans* K1. A Qubit fluorometer (Invitrogen Co., Carlsbad, CA) was used to quantify DNA prior to library preparation with a total DNA input of 1 ng using the Nextera XT DNA Library Preparation Kit and the Nextera Index Kit (Illumina, Inc. San Diego, CA). A proportional amount of Illumina Nextera XT fragmentation enzyme (Illumina) was used to fragment genomic DNA. Combinatory dual indices were added to each sample prior to 12 cycles of PCR to construct libraries. The libraries were purified using AMpure magnetic beads (Beckman Coulter, Inc., Brea, CA) and were eluted in QIAGEN EB buffer (QIAGEN, Inc., Redwood City, CA). DNA libraries were again quantified using a Qubit fluorometer and the Qubit™ dsDNA HS Assay Kit (Invitrogen). Sequencing of the libraries was performed on an Illumina HiSeq platform, 2x250bp (Illumina, Inc.). The raw reads were then processed using MultiQC (v1.11, Seqera Labs, S.L., Barcelona, Spain), and analysis was conducted by sequencing data from all samples (2,172,237,723 total reads) to verify that the read quality met threshold criteria (Phred score >20) and that there was no excessive adapter content. The median number of reads was 18,408,794.26 reads per sample. Sequencing efficacy of all the 118 samples was processed for quality control and showed an average of 54.41% GC content, 151 bp length, and 11.39% failure of modules. The processing of the raw read data was performed in collaboration with CosmosID to map the reads using their custom-curated bacterial, fungal, viral, and antibiotic-resistance genomic database. Classification methods utilized a high-performance data-mining k-mer-based algorithm that disambiguated the millions of short sequence reads into discrete genomes, engendering the particular sequences. To ensure that these generated the expected results, positive and negative internal controls were included. Fungal identification was based on the entire genomes of the organisms referenced in the GenBank™ database (Franzosa et al., 2018). Taxonomic classification methods were performed according to the CosmosID databases of reference genomes that are continuously curated by CosmosID scientists as previously described (Hasan et al., 2014; Lax et al., 2014; Ponnusamy et al., 2016). Abundance scores were calculated by translation of the fine-grain composite k-mer statistics, coverage depth estimation, and genome size information. The relative abundance was calculated by dividing the counts for each taxon by the sum per sample for downstream comparative analysis or differential abundance analysis.

### 2.4 Statistics

The differences in community diversity in the fungal composition of the samples were computed using the CosmosID-HUB Microbiome application. Beta diversity results were compared using permutational multivariate analysis of variance (PERMANOVA) analyses of the relative abundances of species comprising the fungal communities using the Bray–Curtis matrix to test for significance. Visualization of the pairwise dissimilarity matrix generated by Bray-Curtis was conducted by principal coordinate analysis (PCoA). Comparisons of beta diversity indices were done using Bray–Curtis dissimilarity values with 999 permutations on the CosmosID-HUB microbiome analyses platform^2^ with a significance value of *p* < 0.001. The PCoA was performed using JMP® 15.1.0 (SAS Institute Inc., Cary, NC). Characterization of the fungal species driving the differences between two or more fungal communities in biological samples was done using linear discriminant analysis effect size (LEfSe) on the CosmosID-HUB analyses platform. This determines the taxa that best discriminate differences based on relative abundances. This suggests sample biomarkers that best explain the effect-differentiating phenotypes of interest (Segata et al., 2011). The linear discriminant analysis (LDA) scores measured the degree of consistent statistical and biological differences in relative abundances between species in the cohorts, indicating that species biomarkers were found to be discriminative among each cohort. The score was obtained by computing the base_10_ logarithm of the value using a *p*-value of *p* < 0.001 for the comparative factorial Kruskal–Wallis test and by using a high-confidence LDA cut-off of ≥3.3. Venn comparisons of fungal communities in the individual elements (CV manure, CV lagoon, CV trough, CV house fly, CV stable fly, FT manure, FT lagoon, FT trough, FT house fly, and FT stable fly), the combined management systems (CV and FT) and components (manure, lagoon, trough, house fly, and stable fly) of the dairy fungi were conducted using InteractiVenn (Heberle et al., 2015). Only fungi designated to at least the genus level were used in the Venn analysis. The smaller number of FT stable fly samples obtained limit conclusions about management-style differences for this component. This study represents a single-site dairy and a single point of time which limits generalization to other agroecosystems.

## 3 Results

**Figure 1:**
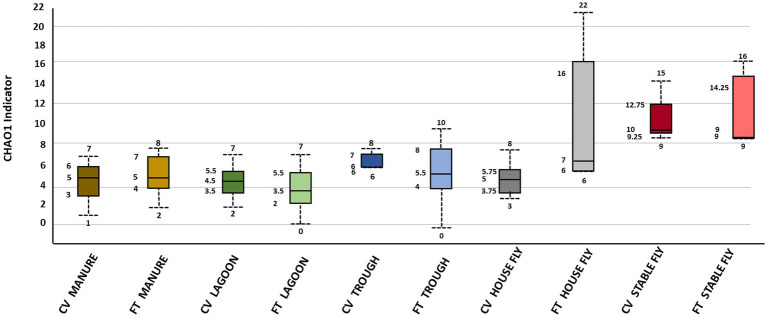
Alpha diversity graphic: box and whiskers diagram of the Chao1 diversity results of the fungal microbiomes of the elements within the dairy components (manure, lagoons, troughs, stable fly, and house fly) separated into results from both dairy management systems of cross-vent (CV) and flow-through (FT) free-stall systems.

A list of the fungal species (*n* = 64) unique to a specified single component or shared by a group of components, when CV and FT free-stall management systems were combined, is supplied in Supplementary Table S1. The CHAO1 alpha diversity estimates of species richness are presented in Figure 1. The stable flies had a CHAO1 indicator, suggesting higher species richness within the samples, and the lagoons had a CHAO1 indicator, suggesting low species richness.

### 3.1 Beta diversity

**Figure 2:**
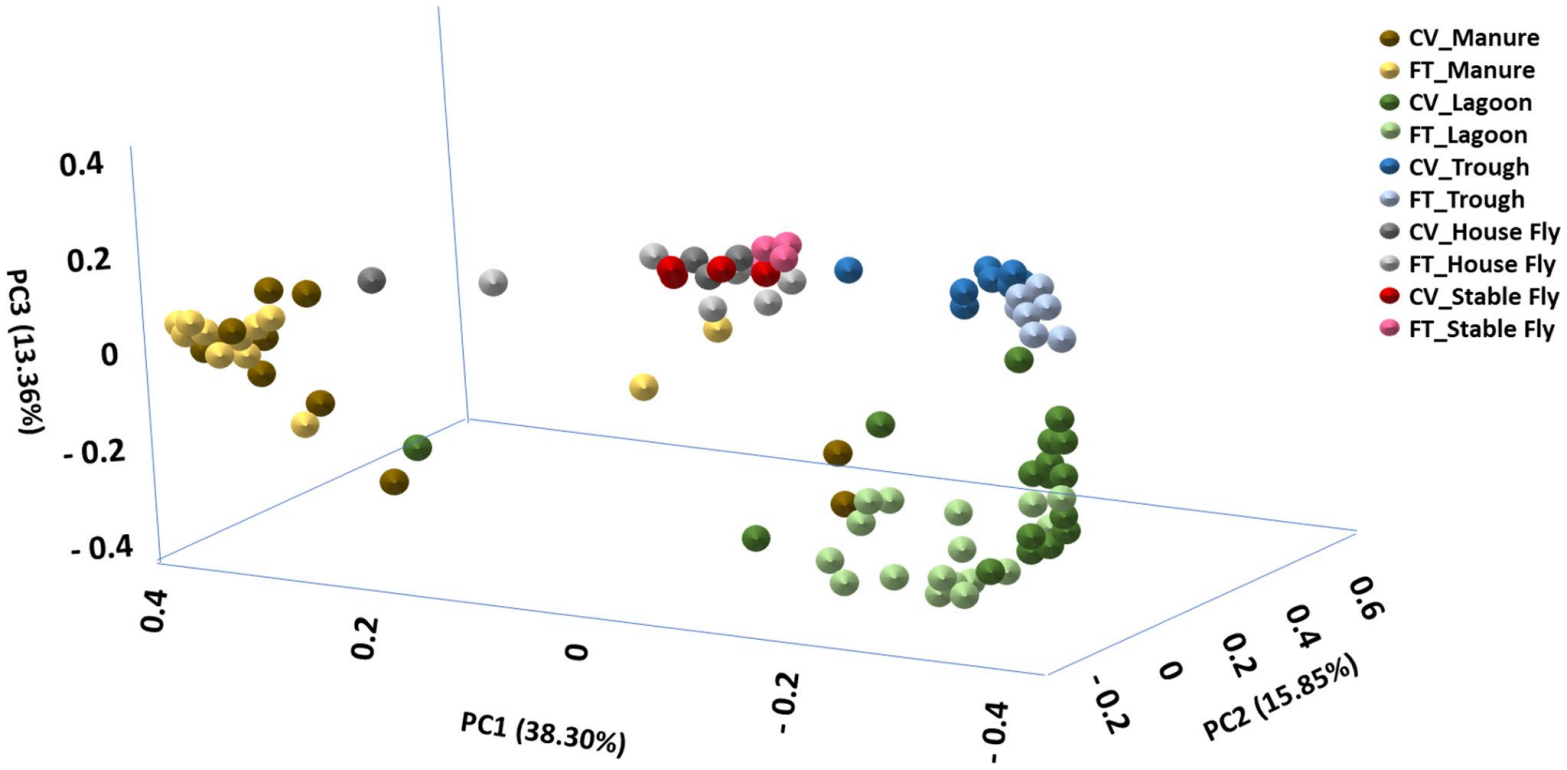
Principal coordinate analysis scatter plot using the Bray–Curtis dissimilarity values of relative abundances of lineages within the dairy fungal communities. Samples were collected from the components listed within the cross-vent (CV) and flow-through (FT) management areas.

The principal coordinate analysis plot using Bray–Curtis dissimilarity values to visually display the distances between the dairy fungal samples is presented (Figure 2). In general, the CV and FT samples collected from the same components clustered together, suggesting similarity. PC1 and PC2 explained over 54.15% of the percentage of variance, suggesting more than 50 percent of the species contributed to the overall dissimilarity.

PERMANOVA statistical comparisons (Table 1) demonstrated that most of the individual components had different species diversity within the same management system. However, species comparisons within the same component between the two different management systems were not different, except between the lagoons and when all components were combined. The combined management system results demonstrated differences in the diversity of the components.

**Table 1:** Fungal diversity comparisons.

**Within Mgt system comparisons**					**Within Mgt system comparisons**				
**CV**	**CV**	***n***	Statistic*	***p*-value**	**FT**	**FT**	***n***	Statistic*	***p*-value**
M	L	40	78.632	**0.001**	M	L	40	42.252	**0.001**
M	T	30	72.347	**0.001**	M	T	30	48.037	**0.001**
M	HF	25	24.173	**0.001**	M	HF	25	15.555	**0.001**
M	SF	25	29.833	**0.001**	M	SF	23	24.064	0.002
L	T	30	19.668	**0.001**	L	T	30	15.308	**0.001**
L	HF	25	27.779	**0.001**	L	HF	25	9.727	**0.001**
L	SF	25	26.634	**0.001**	L	SF	23	10.004	0.002
T	HF	15	25.119	**0.001**	T	HF	15	12.645	**0.001**
T	SF	15	22.993	**0.001**	T	SF	13	16.814	0.003
HF	SF	10	4.003	0.010	HF	SF	8	1.881	0.094
**Combined Mgt system comparisons**					**Between Mgt system comparisons**				
**CV + FT**	**CV + FT**	***n***	**Statistic**	***p*-value**	**CV**	**FT**	***n***	**Statistic**	***p*-value**
M	L	80	95.508	**0.001**	All	All	118	24.614	**0.001**
M	T	60	109.122	**0.001**	M	M	40	1.472	0.217
M	HF	50	35.907	**0.001**	L	L	40	7.167	**0.001**
M	SF	48	47.105	**0.001**	T	T	20	2.689	0.004
L	T	60	25.995	**0.001**	HF	HF	10	0.905	0.517
L	HF	50	26.047	**0.001**	SF	SF	8	2.452	0.046
L	SF	48	25.212	**0.001**					
T	HF	30	31.451	**0.001**					
T	SF	30	32.939	**0.001**					
HF	SF	18	4.149	0.002					

PERMANOVA comparisons using Bray-Curtis indices between individual elements of the management (Mgt) systems, cross-vent (CV) and flow-through (FT), or of the combined management system components, manure (M), lagoon (L), trough (T), house fly (HF) and stable fly (SF).

Significant *p*-values (p ≤ 0.001) are in bold.

* Pseudo F-statistic, calculated by dividing the varition between groups by the variation within the groups then multiplying by group numbers to derive the *p*-value.

### 3.2 Species-specific comparisons

#### 3.2.1 Common species within the overall fungal communities

The top 15 most abundant species at the dairy made up 93.50% of the relative abundance of species (Table 2). No single fungal species was present in every replicate sample analyzed; however, two species, *Brettanomyces bruxellensis* and *Oidium heveae* were present in all dairy components when the results of management systems were combined.

**Table 2:** Top 15 fungal species in each of the dairy elements, components, and management style cohorts and their percent abundance (%) of combined samples.

	**%**	**MANURE**	**%**	**LAGOON**	**%**	**TROUGH**	**%**	**HOUSE FLY**	**%**	**STABLE FLY**
Elements										
Cross vent (CV)	80.13	*Geotrichum candidum*	29.46	*Onygenales sp*	29.18	*Onygenales sp*	34.33	*Nosema bombycis*	27.60	*Diutina catenulata*
	5.68	*Brettanomyces bruxellensis*	28.65	*Brettanomyces bruxellensis*	28.29	*Epichloe sylvatica*	21.43	*Diutina catenulata*	19.83	*Oidium heveae*
	5.08	*Pichia kudriavzevii*	27.16	*Cantharellus sp*	10.16	*Cantharellus sp*	15.63	*Pichia kudriavzevii*	18.04	*Alternaria alternata*
	2.21	*Onygenales sp*	4.77	*Cantharellus cibarius*	8.26	*Clavaria fumosa*	14.34	*Geotrichum candidum*	9.39	*Alternaria tenuissima*
	1.58	*Aspergillus chevalieri*	4.14	*Geotrichum candidum*	5.98	*Morchella eximia*	6.90	*Albatrellus ellisii*	5.97	*Epicoccum nigrum*
	1.20	*Trichosporon sp*	3.67	*Puccinia arachidis*	5.19	*Lentinus polychrous*	2.28	*Alternaria sp*	4.21	*Alternaria sp. MG1*
	1.08	*Morchella eximia*	1.02	*Clavaria fumosa*	3.16	*Pichia kudriavzevii*	1.79	*Enterocytozoon hepatopenaei*	3.86	*Pichia kudriavzevii*
	0.86	*Basipetospora chlamydospora*	0.63	*Blastomyces emzantsi*	3.14	*Fusarium subglutinans*	1.70	*Tricholoma sp. MG99*	3.62	*Geotrichum candidum*
	0.68	*Cantharellus sp*	0.32	*Lentinus polychrous*	2.82	*Cantharellus cibarius*	1.61	*Malassezia sp*	3.16	*Nosema bombycis*
	0.67	*Diutina catenulata*	0.18	*Diutina catenulata*	2.04	*Oidium heveae*			1.29	*Alternaria sp*
	0.31	*Mucor circinelloides*			1.39	*Brettanomyces bruxellensis*			0.83	*Aureobasidium melanogenum*
	0.17	*Trichosporon asahii*			0.22	*Aspergillus sp*			0.62	*Mucor circinelloides*
	0.14	*Oidium heveae*			0.18	*Aspergillus sp. HF37*			0.59	*Alternaria arborescens*
	0.12	*Puccinia arachidis*							0.41	*Enterocytozoon hepatopenaei*
	0.06	*Aspergillus sp*							0.18	*Alternaria gaisen*
Flow through (FT)	77.27	*Geotrichum candidum*	47.00	*Brettanomyces bruxellensis*	39.14	*Onygenales sp*	24.94	*Diutina catenulata*	58.18	*Diutina catenulata*
	4.54	*Thermomyces lanuginosus*	19.88	*Onygenales sp*	20.47	*Cantharellus sp*	14.41	*Pichia kudriavzevii*	19.34	*Pichia kudriavzevii*
	4.00	*Brettanomyces bruxellensis*	18.17	*Cantharellus sp*	16.60	*Epichloe sylvatica*	12.91	*Puccinia arachidis*	10.27	*Alternaria alternata*
	3.63	*Oidium heveae*	4.30	*Puccinia arachidis*	12.57	*Clavaria fumosa*	12.80	*Morchella eximia*	4.19	*Alternaria tenuissima*
	2.65	*Aspergillus chevalieri*	3.14	*Basipetospora chlamydospora*	3.06	*Cantharellus cibarius*	9.90	*Geotrichum candidum*	1.50	*Geotrichum candidum*
	1.94	*Onygenales sp*	2.94	*Diutina catenulata*	2.05	*Pichia kudriavzevii*	9.67	*Nosema bombycis*	1.29	*Alternaria sp. MG1*
	1.67	*Aspergillus sp*	2.03	*Cantharellus cibarius*	1.97	*Aspergillus sp*	2.88	*Alternaria arborescens*	1.22	*Onygenales sp*
	1.49	*Rhizomucor sp*	1.30	*Erysiphe pulchra*	1.44	*Puccinia arachidis*	2.87	*Alternaria alternata*	0.74	*Epicoccum nigrum*
	1.00	*Trichosporon sp*	0.42	*Acremonium chrysogenum*	1.13	*Lentinus polychrous*	1.92	*Alternaria tenuissima*	0.51	*Alternaria arborescens*
	0.67	*Diutina catenulata*	0.41	*Blastomyces emzantsi*	0.80	*Fusarium subglutinans*	1.91	*Alternaria sp*	0.49	*Alternaria sp*
	0.61	*Trichosporon asahii*	0.22	*Sodiomyces alkalinus*	0.78	*Brettanomyces bruxellensis*	1.82	*Oidium heveae*	0.44	*Epichloe sylvatica*
	0.13	*Cantharellus sp*	0.18	*Oidium heveae*			1.73	*Alternaria sp. MG1*	0.34	*Clavaria fumosa*
	0.13	*Lichtheimia sp*					0.78	*Preussia sp. BSL10*	0.26	*Oidium heveae*
	0.12	*Pichia kudriavzevii*					0.48	*Alternaria alternata complex*	0.26	*Metschnikowia sp*
	0.11	*Epichloe sylvatica*					0.32	*Cladosporium sp. SL-16*	0.24	*Diutina rugosa*
Components										
CV + FT	78.70	*Geotrichum candidum*	37.34	*Brettanomyces bruxellensis*	33.90	*Onygenales sp*	23.34	*Diutina catenulata*	39.07	*Diutina catenulata*
	4.84	*Brettanomyces bruxellensis*	24.92	*Onygenales sp*	22.75	*Epichloe sylvatica*	20.88	*Nosema bombycis*	15.13	*Alternaria alternata*
	2.60	*Pichia kudriavzevii*	22.90	*Cantharellus sp*	15.04	*Cantharellus sp*	14.96	*Pichia kudriavzevii*	12.49	*Oidium heveae*
	2.27	*Thermomyces lanuginosus*	3.97	*Puccinia arachidis*	10.30	*Clavaria fumosa*	11.92	*Geotrichum candidum*	9.66	*Pichia kudriavzevii*
	2.12	*Aspergillus chevalieri*	3.47	*Cantharellus cibarius*	3.26	*Lentinus polychrous*	7.04	*Puccinia arachidis*	7.44	*Alternaria tenuissima*
	2.08	*Onygenales sp*	2.18	*Geotrichum candidum*	3.15	*Morchella eximia*	6.98	*Morchella eximia*	4.01	*Epicoccum nigrum*
	1.88	*Oidium heveae*	1.49	*Diutina catenulata*	2.93	*Cantharellus cibarius*	3.13	*Albatrellus ellisii*	3.12	*Alternaria sp. MG1*
	1.10	*Trichosporon sp*	1.49	*Basipetospora chlamydospora*	2.63	*Pichia kudriavzevii*	2.08	*Alternaria sp*	2.82	*Geotrichum candidum*
	0.86	*Aspergillus sp*	0.61	*Erysiphe pulchra*	2.03	*Fusarium subglutinans*	1.57	*Alternaria arborescens*	1.98	*Nosema bombycis*
	0.74	*Rhizomucor sp*	0.54	*Clavaria fumosa*	1.10	*Brettanomyces bruxellensis*	1.57	*Alternaria alternata*	0.99	*Alternaria sp*
	0.67	*Diutina catenulata*	0.52	*Blastomyces emzantsi*	1.07	*Oidium heveae*	1.05	*Alternaria tenuissima*	0.56	*Alternaria arborescens*
	0.54	*Morchella eximia*	0.20	*Acremonium chrysogenum*	1.05	*Aspergillus sp*	0.99	*Oidium heveae*	0.52	*Aureobasidium melanogenum*
	0.43	*Basipetospora chlamydospora*	0.17	*Lentinus polychrous*	0.68	*Puccinia arachidis*	0.94	*Alternaria sp. MG1*	0.46	*Onygenales sp*
	0.41	*Cantharellus sp*	0.10	*Sodiomyces alkalinus*	0.10	*Aspergillus sp. HF37*	0.81	*Enterocytozoon hepatopenaei*	0.39	*Mucor circinelloides*
	0.39	*Trichosporon asahii*	0.08	*Oidium heveae*			0.77	Tricholoma sp. MG99	0.26	*Enterocytozoon hepatopenaei*

	**%**	**Cross Vent**	**%**	**Flow Through**	%	Dairy
Management systems						
Combined Components	29.59	*Geotrichum candidum*	28.74	*Pichia kudriavzevii*	29.18	*Geotrichum candidum*
	15.42	*Onygenales sp*	16.67	*Aspergillus sp*	14.46	*Onygenales sp*
	11.67	*Brettanomyces bruxellensis*	13.44	*Mucor circinelloides*	14.09	*Brettanomyces bruxellensis*
	10.97	*Cantharellus sp*	9.19	*Byssochlamys sp. IMV 00045*	10.11	*Cantharellus sp*
	4.72	*Epichloe sylvatica*	6.97	*Meyerozyma sp. JA9*	5.63	*Diutina catenulata*
	4.37	*Diutina catenulata*	3.00	*Albatrellus ellisii*	3.76	*Epichloe sylvatica*
	3.84	*Pichia kudriavzevii*	2.95	*Cantharellus cibarius*	3.41	*Pichia kudriavzevii*
	3.12	*Nosema bombycis*	2.73	*Cyberlindnera jadinii*	2.12	*Nosema bombycis*
	2.06	*Cantharellus cibarius*	2.04	*Ascodesmis nigricans*	2.10	*Puccinia arachidis*
	2.04	*Oidium heveae*	1.62	*Trichosporon asahii*	1.87	*Clavaria fumosa*
	1.72	*Clavaria fumosa*	1.56	*Fusarium subglutinans*	1.81	*Oidium heveae*
	1.50	*Alternaria alternata*	1.37	*Epichloe sylvatica*	1.62	*Cantharellus cibarius*
	1.36	*Morchella eximia*	1.15	*Thermomyces lanuginosus*	1.36	*Morchella eximia*
	1.27	*Puccinia arachidis*	1.04	*Calonectria naviculata*	1.20	*Alternaria alternata*
	0.97	*Lentinus polychrous*	1.01	*Aspergillus chevalieri*	0.78	*Thermomyces lanuginosus*

Indicator species analyses by LEfSe score were used to explore the taxa that best discriminated the fungal composition between the various cohorts (Table 3). The analyses describe three main outputs comparing the differences among the composition of fungal communities in (1) the CV and FT areas; (2) the elements (CV manure, CV lagoon, CV trough, CV house Fly, CV stable Fly, FT manure, FT lagoon, FT trough, FT house Fly, and FT stable Fly); and (3) the components (manure, lagoon, trough, house fly, and stable fly). The flies had the most indicator species, indicating a broad and unique fungal community in comparison to the other components. Whereas the lagoons had the fewest indicator species.

**Table 3:** Linear discriminant analysis effect size (LEfSe) output for each of the dairy elements, components, and management style cohorts.

	**LDA**	**MANURE**	**LDA**	**LAGOON**	**LDA**	**TROUGH**	**LDA**	**HOUSE FLY**	**LDA**	**STABLE FLY**
Elements										
Cross vent (CV)	5.60	*Geotrichum candidum*	5.14	*Cantharellus sp.*	5.16	*Epichloe sylvatica*	5.25	*Nosema bombycis*	5.01	*Alternaria alternata*
					4.43	*Lentinus polychrous*	4.44	*Albatrellus ellisii*	4.86	*Oidium heveae*
							4.04	*Alternaria sp.*	4.76	*Alternaria tenuissima*
							3.98	*Enterocytozoon hepatopenaei*	4.54	*Epicoccum nigrum*
							3.85	*Tricholoma sp.* MG99	4.40	*Alternaria sp.* MG1
							3.85	*Malassezia sp.*	3.75	*Aureobasidium melanogenum*
									
Flow through (FT)	4.25	*Thermomyces lanuginosus*	5.33	*Brettanomyces bruxellensis*	5.25	*Onygenales sp*	4.82	*Morchella eximia*	5.49	*Diutina catenulata*
	4.16	*Aspergillus chevalieri*			4.76	*Clavaria fumosa*	4.82	*Puccinia arachidis*	4.99	*Pichia kudriavzevii*
	3.97	*Rhizomucor sp.*					4.12	*Alternaria arborescens*		
							3.68	*Preussia sp. BSL10*		
							3.46	*Alternaria alternata complex*		
Components										
Combined CV + FT	5.60	*Geotrichum candidum*	5.25	*Brettanomyces bruxellensis*	5.19	*Onygenales sp.*	5.11	*Nosema bombycis*	5.30	*Diutina catenulata*
	3.98	*Aspergillus chevalieri*	5.03	*Cantharellus sp.*	5.05	*Epichloe sylvatica*	4.89	*Pichia kudriavzevii*	4.88	*Alternaria alternata*
	3.67	*Trichosporon sp.*			4.67	*Clavaria fumosa*	4.47	*Puccinia arachidis*	4.78	*Oidium heveae*
					4.20	*Lentinus polychrous*	4.24	*Albatrellus ellisii*	4.56	*Alternaria tenuissima*
							3.97	*Alternaria arborescens*	4.30	*Epicoccum nigrum*
							3.96	*Alternaria sp.*	4.22	*Alternaria sp.* MG1
							3.85	*Enterocytozoon hepatopenaei*	3.62	*Aureobasidium melanogenum*
							3.65	*Preussia sp.* BSL10		

	**LDA**	**CV**	**LDA**	**FT**
Management systems				
Combined Components	3.66	*Lentinus polychrous*	4.04	*Thermomyces lanuginosus*
	3.36	*Enterocytozoon hepatopenaei*	3.53	*Aspergillus chevalieri*
	3.31	*Mucor circinelloides*	3.53	*Rhizomucor sp.*

Differentially abundant fungi with linear discriminant analysis (LDA) scores ≥3.3. The output of the analyses determines the species likely to give discriminative indication between the cohorts using the comparative factorial Kruskal-Wallis test at a statistical significance of p > 0.01.

#### 3.2.2 Comparisons of species in cohorts

Venn comparisons by the management system of the composition of species in each component were conducted. A total of 64 species of fungi were identified within the elements of the dairy sampled and compared by management system (Table 4), by element (Table 5), and by component (Table 6).

**Table 4:** Two-way Venn comparisons of management system species.

Components	Unique to cross-vent	Shared	Unique to flow-through
	#1		#2
All	10 (15.6)*	30 (46.9)	24 (37.5)
Manure	5 (23.8)	11 (52.4)	5 (23.8)
Lagoon	3 (20.0)	7 (46.7)	5 (33.3)
Trough	3 (21.4)	10 (71.4)	1 (7.1)
House fly	4 (11.1)	5 (13.9)	27 (75)
Stable fly	8 (25.8)	12 (38.7)	11 (35.5)
Fly (house + stable)	8 (17.4)	14 (30.4)	24 (52.2)

*The number and (percentage) of species.

**Table 5:** Five-way Venn comparisons of elements by management system species.

		**Mgt system**					
		**CV**		**FT**		**Dairy**	
**COMPONENT**		Unique*	**Total†**	**Unique**	**Total**	**Unique**	**Total**
#1	MANURE	5 (12.5)‡	16	7 (13.0)	16	8 (12.5)	21
#2	LAGOON	1 (2.5)	10	5 (9.3)	12	4 (6.3)	15
#3	TROUGH	3 (7.5)	13	2 (3.7)	11	2 (3.1)	14
#4	HOUSE FLY	2 (5.0)	9	14 (25.9)	32	13 (20.3)	36
#5	STABLE FLY	11 (27.5)	20	2 (3.7)	23	5 (7.8)	31
		Shared	Total	Shared	Total	Shared	Total
All Components		0 (0.0)	40	1 (1.9)	54	2 (3.1)	64

^*^ species unique to only that element.

^†^ total number of species in that element.

^‡^ the number (percentage) of species.

Mgt, management; CV, cross-vent; FT, flow-through; Dairy, CV + FT.

**Table 6:** Two-way Venn comparisons of component species.

Components		Unique to #1	Shared	Unique to #2
#1	#2			
Manure	Lagoon	13 (46.4)*	8 (28.6)	7 (25.0)
	Trough	12 (46.2)	9 (34.6)	5 (19.2)
	House fly	14 (28.0)	7 (14.0)	29 (58.0)
	Stable fly	12 (27.9)	9 (20.9)	22 (51.2)
Lagoon	Trough	7 (33.3)	8 (38.1)	6 (28.6)
	House fly	10 (21.7)	59 (10.9)	31 (67.4)
	Stable fly	8 (20.5)	7 (17.9)	24 (61.5)
Trough	House fly	9 (20.0)	5 (11.1)	31 (68.9)
	Stable fly	7 (18.4)	7 (18.4)	24 (63.2)
House fly	Stable fly	15 (32.6)	21 (45.7)	10 (21.7)

*Number and (percentage) of species.

The comparisons revealed that of the 64 species present, 30 were shared between the barns. FT barns had the most unique species (24) between the two barns (Table 4). Of the individual components, the flies (combined stable and house) carried the greatest number of shared species (14). The troughs had the least number of species unique to the CV (3) and FT barns (1), but a higher number of shared species (10).

When both the management systems’ data were combined to represent the entire dairy fungal community, the house flies carried the greatest number of species (36), 9 of which were found in the CV barn and 32 in the FT barn (Table 5). Thirteen species were not shared with the other components at the dairy, 2 species were unique to the house flies in the CV barn, and 14 species were unique to house flies in the FT barn. The combined trough samples carried the least number of species (14), 13 species were found in the CV barn, and 11 species were found in the FT barn. Two species were not shared with the other components at the dairy, 3 were unique to the troughs in the CV barn, and 2 were unique to the troughs in the FT barn. Of the species, two (3.1%) were found in all components of the dairy (CV and FT combined). One (1.9%) species was found in all components of the FT area, whereas none were shared by all five components within the CV area.

The house flies and stable flies shared the most species, 21 (45.7%), in paired comparisons of the components (combined management systems) (Table 6). The fly species represented different feeding approaches and associated physiological structures and gut systems; however, they showed extensive overlap of fungal species. The second most shared fungal communities were between the manure and stable flies, which shared nine (20.9%) of their fungal species, as well as the manure and trough, which shared nine (34.6%) species. Interestingly, the house fly shared the least number of fungal species with each of the other components, manure, lagoons, and troughs: seven (14.0%), five (10.9%), and five (11.1%), respectively.

### 3.3 Taxa of fungi

**Figure 3:**
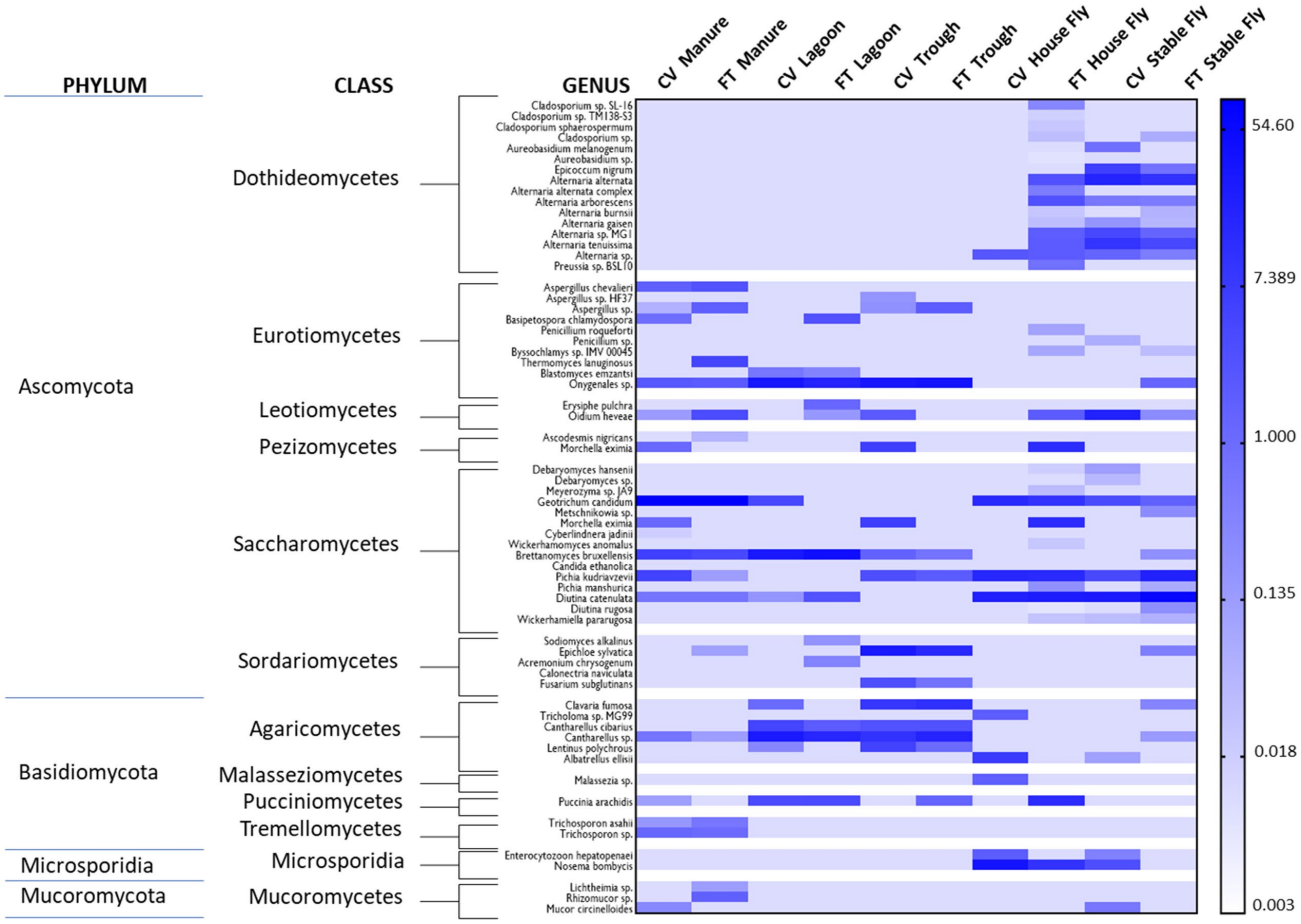
Heatmap of the percent relative abundance of fungi identified to the phylum, class, and species levels within the components (manure, lagoon, trough, house fly, and stable fly) of the cross-vent (CV) and flow-through (FT) free-stall management systems. For natural log transformation, “0” was converted to “0.001”.

Species from 4 phyla and 12 classes of fungi were identified (# species / % relative abundance of total 118 samples): Saccharomycetes (14/52.35%), Eurotiomycetes (10/17.28%), Agaricomycetes (6/14.57%), Sordariomycetes (5/4.19%), Dothideomycetes (16/2.98%), Microsporidia (2/2.21%), Pucciniomycetes (1/2.10%), Leotiomycetes (2/2.01%), Pezizomycetes (2/1.37%), Tremellomycetes (2/0.51%), Mucoromycetes (3/0.36%), and Malasseziomycetes (1/0.07%) (Figure 3).

## 4 Discussion

Fungi are commonly found in dairies due to the abundance of suitable environmental niches, particularly in high moisture areas (Garnier et al., 2017). Unintended fungal contaminants in dairy products can also lead to off-odor and off-flavor and subsequent economic losses of dairy products (Filtenborg et al., 1996). Those that produce mycotoxins can pose significant risks to human food safety, as well as to the health of the cattle and their fetuses (Applebaum et al., 1982; Cortese, 2009; Jiang et al., 2021; Vlasova and Saif, 2021). Fungi are difficult to categorize as fully pathogenic, innocuous, or even beneficial due to the multitude of secondary metabolites they can produce while attempting to control their own environment. Those that are pathogenic can also produce beneficial enzymes and other useful molecular components exploited for antimycotic, antiparasitic, and antibacterial purposes, anticancer therapies, industrial uses, etc. (Eagan and Keller, 2025). Additionally, species that are commonly innocuous can become opportunistic pathogens depending on their load and the immune status of the host plant or animal with which they interact. Therefore, the categorization of the fungi discussed below as pathogenic or beneficial is artificial inasmuch as it is dependent on the existing environmental conditions. Additionally, some species have not been well studied for their beneficial or harmful effects. Overall, of the 64 species identified, 14 species were present in ≥ 1.0% relative abundance and 18 species were present in ≤ 0.01% relative abundance. Taking the overlapping characteristics into account, eleven fungi presented a definitive risk due to the secondary production of mycotoxins that could be detrimental to animal health. However, these represented only 3.10% relative abundance. Another 16 species (32.72% relative abundance) were known opportunistic pathogens. Fifteen (30.34%) were known plant pathogens. Twenty-six (29.93%) had reported beneficial effects or uses. Seven (3.89%) were innocuous or unstudied/reported species.

Fungi can enter the system unintentionally, such as through plants used for cattle feed, during storage of silage, through water sources, and through animals (Franchino et al., 2024). On the flip side, there are beneficial fungal species that are deliberately added to various foods for flavoring, manufacturing cheese, fermenting dairy products, or as pre- and probiotics (Buerth et al., 2016; Garnier et al., 2017; Angulo et al., 2020; Zhang et al., 2022; Chu et al., 2023; Kamilari et al., 2023; Panya et al., 2024). Additionally, some anaerobic fungi are critical microbes in the rumen of cattle. These are needed to produce enzymes, such as cellulases and xylanases, that assist in breaking down tough fibers and the proper functioning of the rumen for digestion of forages (Matthews et al., 2019; Wang et al., 2022).

The different dairy components sampled in this study represented diverse habitats for fungi. The troughs contained water with some organic sediments, but they were considerably less than in comparison with the other components sampled. The manure obviously had less moisture than the troughs but was higher in organic materials. Although a major component of a lagoon is manure, it also receives urine, bedding materials, feed, cattle hair, wash water, etc., and therefore exhibits its own very distinctive conditions. Anaerobic lagoons, such as those in this dairy, have high organic content, near-neutral pH, and aerobic and temperature conditions varying with depth (Bella and Rao, 2023).

The four most abundant fungi found at this dairy (*Geotrichum candidum*, *Onygenales* sp., *Brettanomyces bruxellensis*, and *Cantharellus* sp.) made up 68% of the relative abundance of fungi. No one species of fungus was present in all elements, but *Brettanomyces bruxellensis* and *Oidium heveae* were present in all components (management systems combined). *Brettanomyces bruxellensis* occurs naturally on the skins of fruit that can be fed to cattle, and it can cause spoilage if spread to dairy products (Smith and Divol, 2016; Luo et al., 2021). Analysis showed that *Brettanomyces bruxellensis* was a discriminatory species for the lagoons. This is likely because 71% of its occurrence was in the lagoons, where its ability to survive in harsh environments would be an advantage (Smith and Divol, 2016). *Oidium heveae* is pathogenic to many plants and probably entered the system via the cattle feed and silage, and spread from there (Zhai et al., 2021).

We compared the number of different species in the various samples to determine where, in the dairy, species overlap occurred. Such an overlap would imply a possible path for their spread. Results did not show significant differences in diversity between management systems, except in the lagoons. There were, however, significant differences between most components. This indicated a lack of sharing of many fungal species between components, but not all. But this information could be important for focused pathogen control.

The house flies carried the greatest number of unique species in the FT barns, and the stable flies carried the greatest number in the CV barns. The flies specifically represent specialized feeding behaviors: the hematophagous blood-feeding stable flies and the omnivorous sponge-feeding house flies. This would impact their choice of locations to visit within the barns and thus influence their overall exposure to various fungi (Scully et al., 2017; Ysquierdo et al., 2017; Park et al., 2019). The stable fly would likely encounter similar fungal species on the cattle from which they feed, whether in the CV or FT barn. While adult stable flies are very mobile, they tend to feed only once per day and then spend most of their time resting in the shade at on-farm locations, not on the animal (Hogsette et al., 1989; Showler and Osbrink, 2015). Alternatively, the omnivorous house flies acquire food from many sources and are described to move in short, disjointed, circuitous flights around agricultural facilities (Gerry et al., 2011; Geden et al., 2021). Both fly species are therefore exposed to many locations within the dairy that support fungi (Hogsette et al., 1989). The house flies from the FT barn had the most unique species compared to the flies from the CV barns. Perhaps the wider variety of resource types utilized by sponge feeders is available for foraging in the FT barn, which is more open to the outside environment. The microsporidia, *Nosema bombycis* was the most discriminative fungi for the house fly by LEfSe analysis. Notably, it can cause microsporidiosis in mammals at a significant economic loss to producers and is a threat to public health (Stentiford et al., 2016). *Diutina catenulata* and *Alternaria alternata* were the most discriminative fungi for the stable fly by LEfSe analysis.

The most abundant potential mammalian pathogens were found exclusively in the flies. However, the relative abundance of mammalian fungal pathogens at this dairy was low, at only 3.10%. Although the two fly species represented different feeding approaches and associated physiological structures, they showed extensive overlap in their carriage of fungal species. The homeostasis and metabolism of host nutrients in the gut of the insect are known to utilize fungal enzymes (Khan et al., 2020; Li et al., 2023). It has been noted that the internal microbial community of flies is likely more influenced by fly physiology, while the external community is more influenced by the environment (Park et al., 2019). In this experiment, the flies were analyzed as a whole, internal plus external. Of the fungi found exclusively on flies, the class Dothideomycetes stands out. Class Dothideomycetes fungi have been previously associated with house flies from various agricultural locations, such as cattle ranches (Park et al., 2019). Within the class, the genus *Alternaria* are some of the most common fungi encountered in the environment and can enter a dairy operation through feed. As a group, they produce more than 70 secondary metabolites with notable toxicity in animals and therefore can pose a risk to food safety, as well as product quality on the dairies (Escrivá et al., 2017; DeMers, 2022; Xing et al., 2022).

In manure, the most abundant fungi were *Geotrichum candidum* and *Brettanomyces bruxellensis*. *Geotrichum candidum* was the most discriminative fungus for manure by indicator species analysis. It has been found in dairy silage. So if diseased plants were used, it likely spreads into the cattle manure, the lagoons, and the house and stable flies from there (O'Brien et al., 2005). *Pichia kudriavzevii*, an indigenous rumen yeast, is common to cow manure, bedding, on the teats, and in raw milk at dairy facilities (Fernandes et al., 2019; Verdier-Metz et al., 2023). It is interesting that it was not found in the lagoons, given that it is indigenous to cattle manure and has an inherent tolerance to a range of environmental conditions, such as hyperosmotic stress, extreme pH, temperature, or ethanol, not uncommonly found in lagoon environments (Nieto-Sarabia et al., 2022; Chu et al., 2023). The manure and lagoons shared the second-highest number of species and potential pathogens between them. This is not surprising considering that the manure is flushed into the lagoons. However, environmental conditions, such as oxygen, pH, and temperature, would differ between these two components. Lagoons characteristically have minimal to oxygen-free conditions, are slightly acidic and then progress to slightly basic pH, and consist of temperatures between 30 °C and 35 °C (Bharati and Shinkar, 2013; Ma et al., 2023). They are also high in lactose and organic content (Bharati and Shinkar, 2013; Ma et al., 2023). Typically, only anaerobic organisms thrive at depth within the lagoons, and aerobic organisms survive at or near the lagoon surface (McGarvey et al., 2007; Ma et al., 2023). Waste treatment lagoons are influenced by ambient conditions and, in Texas, during the summer, temperatures can exceed 30 °C (Hamilton et al., 2006; Becker et al., 2016). Such elevated temperatures may favor thermophilic fungal species within the dairy environment. The presence of *Thermomyces lanuginosus* in manure was therefore not unexpected, as it is a thermophilic ruminant fungus capable of withstanding elevated temperatures. It produces lignolytic enzymes that help degrade non-starch polysaccharides and increase ruminal volatile fatty acid concentrations, thereby improving energy metabolism and milk yield (Jurkovich et al., 2002). Additionally, it plays a central role in the decomposition of cattle manure (Sun et al., 2022).

At this dairy, analyses showed a difference in lagoon species composition by management system. The two different lagoon systems were managed differently, as noted in the materials and methods. The CV lagoons were filled via the wash waterways, while the FT lagoon received input of manure manually by trucking and received less wash water. Mechanical aeration was not provided to their lagoons. The lagoons and manure shared many species; however, some species differed presumably because of the distinctive environmental conditions associated with the two components. *Onygenales* sp. are found in decomposing organic matter around animal dwellings and were identified in manure, lagoons, and troughs at this dairy. It can digest keratin, and this genus contains species that are common pathogens of animal hair, nail, and skin (Chaturvedi and de Hoog, 2020). Although it has previously been reported to be carried by house flies around penned cattle, at this dairy, it was only found in the CV stable flies. Stable flies would be in contact with the hair, nail, and skin of cattle, suggesting a possible pathway of spread (Ysquierdo et al., 2017). At this dairy, however, the lagoons were not a reservoir of major fungal pathogens.

The most prevalent fungal species in the austere environment of the troughs were also of the genus *Onygenales* sp., followed by the innocuous plant endophyte *Epichloe sylvatica*, and the edible fungi *Cantharellus* sp., *Clavaria fumosa*, and *Lentinus polychrous* (Meijer and Leuchtmann, 1999; Elkhateeb et al., 2021; Fabros et al., 2022). These fungi most likely entered the system through silage or hay and were spread to the troughs by cattle usage. In general, the troughs did not appear to be a reservoir of fungal pathogens.

The cattle health records are presented in the materials and methods section for general information; no direct fungal cause is implied, nor was it measured. The metagenomic DNA sequence data do not distinguish live from dead organisms. Therefore, no conclusions can be drawn as to the virulence of the identified fungi. Additionally, these results focus only on fungal microorganisms while other possible causal microorganisms exist at this dairy (Crippen et al., 2023). But the health records do give an overall view of the issues occurring in relation to the presence of known pathogenic fungi in different components at the facilities. We recognize and strongly advocate that the mere presence of a known pathogen does not translate directly into pathology. Infection would depend on a multitude of confounding factors, such as the likelihood of exposure to and contact with a transmissible concentration of the pathogen, as well as the animal’s general health, as many of the fungi identified are opportunistic pathogens. Therefore, a cow’s immunocompetency would play a significant role in whether morbidity or mortality occurs. Finally, the cattle at this dairy were on a health program and received medications against infectious bovine rhinotracheitis, bovine respiratory syncytial virus, bovine respiratory disease, parainfluenza, coronavirus, leptospirosis, blackleg, enterotoxemia, mastitis, causing coliform Gram-negative bacteria, scours, bovine viral diarrhea, and pinkeye. The dairy health records show that the incidence of disease and other complications at this dairy was low, and this was likely attributed to their fastidious management and health program.

## 5 Conclusion

In this study, we assessed the fungi present in different dairy components (manure, lagoons, and troughs) and flies with different feeding behaviors (house and stable flies) in barns operated with differing management systems (CV and FT). Although many samples were taken at this dairy, the results reported here must be taken in the context that only a very small percentage of niches and the substrates available for fungi to exist were sampled. Diversity comparisons showed that the CV and FT barns were not significantly different in their fungal species richness except between the lagoons. But the fungal composition between the components of the dairy differed from each other, likely due to their unique abiotic and biotic characteristics. The plant pathogens, *Brettanomyces bruxellensis* and *Oidium heveae*, were the only fungi present in each of the dairy components. If it is assumed that these two pathogens entered the system through the feed and silage, they then spread to the manure, lagoons, troughs, and flies, possibly by direct contact with feed, by the cattle, by the flies, by humans, or mechanical means, or a combination of these pathways.

The house and stable flies shared the most species of fungi and carried the most potential mammalian pathogens. It is interesting that the pathogenic species found associated with the flies were not significantly shared with the other components. Therefore, it is unclear whether the pathogens are being efficiently exchanged, either mechanically or biologically. The most opportunistic pathogenic species were identified in the manure. Most plant pathogens and beneficial species were in the lagoons. The most innocuous or understudied species were found in the troughs. These results allow dairy managers to consider advantageous management systems and how to focus pathogenic fungal mitigation efforts on appropriate sites within the dairy operation. Further studies are needed to include sampling of multiple dairies and other dairy sites, such as the cattle, the milking parlor, silage, equipment, and feed. Longitudinal studies would also be required to describe the seasonality of the occurrence of fungi and the impact of the many abiotic confounding factors and different farm management practices. The information from this study will help inform targeted studies to determine the efficacy of any mitigation applied to manipulate these communities.

## Impact statement

Certain fungi at dairy production facilities can cause food safety and animal health issues, but reservoir locations for beneficial and pathogenic fungi have not been extensively explored. Samples collected from dairy manure, lagoons, troughs, and flies at a dairy implementing different management systems were analyzed by shotgun metagenomics, and flies were found to carry more potential mammalian pathogenic species than manure, lagoons, and troughs. Results will help dairy managers to consider advantageous management systems and focus on fungal mitigation efforts at appropriate locations within the dairy.

## Data Availability

The datasets presented in this study can be found in online repositories. The names of the repository/repositories and accession number(s) can be found in the article/Supplementary material.

## Footnotes

1
https://world-weather.info/

2
https://cosmos-hub.com/
