# Supplementary material for: Capturing the fungal diversity in manure, lagoons, troughs, and flies at a commercial dairy
Source: Front Microbiol. 2026 Apr 13;17:1794875. doi: 10.3389/fmicb.2026.1794875 (PMC13112536; doi:10.3389/fmicb.2026.1794875)
Supplement: Supplementary file 1 [file Data_Sheet_1.PDF]

Table S1. Fungi found within the specified single or shared by the collective components of combined cross-vent and flow-through free stall microbiome results.

Components

MANURE	LAGOON	TROUGH	HOUSE FLY	STABLE FLY
21	15	14	36	31
Ascodesmis nigricans	Acremonium chrysogenum	Aspergillus sp. HF37	[Candida] ethanolica	Albatrellus ellisii
Aspergillus chevalieri	Basipetospora chlamydospora	Aspergillus sp	Albatrellus ellisii	Alternaria alternata
Aspergillus sp	Blastomyces emzantsi	Brettanomyces bruxellensis	Alternaria alternata	Alternaria arborescens
Basipetospora chlamydospora	Brettanomyces bruxellensis	Cantharellus cibarius	Alternaria alternata complex	Alternaria burnsii
Brettanomyces bruxellensis	Cantharellus cibarius	Cantharellus sp	Alternaria arborescens	Alternaria gaisen
Cantharellus sp	Cantharellus sp	Clavaria fumosa	Alternaria burnsii	Alternaria sp. MG1
Cyberlindnera jadinii	Clavaria fumosa	Epichloe sylvatica	Alternaria gaisen	Alternaria tenuissima
Diutina catenulata	Diutina catenulata	Fusarium subglutinans	Alternaria sp. MG1	Alternaria sp
Epichloe sylvatica	Erysiphe pulchra	Lentinus polychrous	Alternaria tenuissima	Aureobasidium melanogenum
Geotrichum candidum	Geotrichum candidum	Morchella eximia	Alternaria sp	Brettanomyces bruxellensis
Lichtheimia sp	Lentinus polychrous	Oidium heveae	Aureobasidium sp	Byssochlamys sp. IMV 00045
Morchella eximia	Oidium heveae	Onygenales sp	Brettanomyces bruxellensis	Cantharellus sp
Mucor circinelloides	Onygenales sp	Pichia kudriavzevii	Byssochlamys sp. IMV 00045	Cladosporium sp
Oidium heveae	Puccinia arachidis	Puccinia arachidis	Calonectria naviculata	Clavaria fumosa
Onygenales sp	Sodiomyces alkalinus		Cladosporium sp. SL-16	Debaryomyces hansenii
Pichia kudriavzevii			Cladosporium sp. TM138-S3	Debaryomyces sp
Puccinia arachidis			Cladosporium sphaerospermum	Diutina catenulata
Rhizomucor sp			Cladosporium sp	Diutina rugosa
Thermomyces lanuginosus			Debaryomyces hansenii	Enterocytozoon hepatopenaei
Trichosporon asahii			Diutina catenulata	Epichloe sylvatica
Trichosporon sp			Diutina rugosa	Epicoccum nigrum
			Enterocytozoon hepatopenaei	Geotrichum candidum
			Geotrichum candidum	Metschnikowia sp
			Malassezia sp	Mucor circinelloides
			Meyerozyma sp. JA9	Nosema bombycis
			Morchella eximia	Oidium heveae
			Nosema bombycis	Onygenales sp
			Oidium heveae	Penicillium sp
			Penicillium roqueforti	Pichia kudriavzevii
			Pichia kudriavzevii	Pichia manshurica
			Pichia manshurica	Wickerhamiella pararugosa
			Preussia sp. BSL10	
			Puccinia arachidis	
			Tricholoma sp. MG99	
			Wickerhamiella pararugosa	
			Wickerhamomyces anomalus	

2-way

MANURE<br>LAGOON	MANURE<br>TROUGH	MANURE<br>HOUSE FLY	MANURE<br>STABLE FLY	LAGOON<br>TROUGH	LAGOON<br>HOUSE FLY	LAGOON<br>STABLE FLY	TROUGH<br>HOUSE FLY	TROUGH<br>STABLE FLY	HOUSE FLY<br>STABLE FLY
8	9	7	9	8	5	7	5	7	21
Basipetospora chlamydospora	Aspergillus sp	Brettanomyces bruxellensis	Brettanomyces bruxellensis	Brettanomyces bruxellensis	Brettanomyces bruxellensis	Brettanomyces bruxellensis	Brettanomyces bruxellensis	Brettanomyces bruxellensis	Albatrellus ellisii
Brettanomyces bruxellensis	Brettanomyces bruxellensis	Diutina catenulata	Cantharellus sp	Cantharellus cibarius	Diutina catenulata	Cantharellus sp	Morchella eximia	Cantharellus sp	Alternaria alternata
Cantharellus sp	Cantharellus sp	Geotrichum candidum	Diutina catenulata	Cantharellus sp	Geotrichum candidum	Clavaria fumosa	Oidium heveae	Clavaria fumosa	Alternaria arborescens
Diutina catenulata	Epichloe sylvatica	Morchella eximia	Epichloe sylvatica	Clavaria fumosa	Oidium heveae	Diutina catenulata	Pichia kudriavzevii	Epichloe sylvatica	Alternaria burnsii
Geotrichum candidum	Morchella eximia	Oidium heveae	Geotrichum candidum	Lentinus polychrous	Puccinia arachidis	Geotrichum candidum	Puccinia arachidis	Oidium heveae	Alternaria gaisen
Oidium heveae	Oidium heveae	Pichia kudriavzevii	Mucor circinelloides	Oidium heveae		Oidium heveae		Onygenales sp	Alternaria sp. MG1
Onygenales sp	Onygenales sp	Puccinia arachidis	Oidium heveae	Onygenales sp		Onygenales sp		Pichia kudriavzevii	Alternaria tenuissima
Puccinia arachidis	Pichia kudriavzevii		Onygenales sp	Puccinia arachidis					Alternaria sp
	Puccinia arachidis		Pichia kudriavzevii						Brettanomyces bruxellensis
									Byssochlamys sp. IMV 00045
									Cladosporium sp
									Debaryomyces hansenii
									Diutina catenulata
									Diutina rugosa
									Enterocytozoon hepatopenaei
									Geotrichum candidum
									Nosema bombycis
									Oidium heveae
									Pichia kudriavzevii
									Pichia manshurica
									Wickerhamiella pararugosa

3-way

MANURE<br>LAGOON<br>TROUGH	MANURE<br>LAGOON<br>HOUSE FLY	MANURE<br>LAGOON<br>STABLE FLY	MANURE<br>TROUGH<br>HOUSE FLY	MANURE<br>TROUGH<br>STABLE FLY	MANURE<br>HOUSE FLY<br>STABLE FLY	LAGOON<br>TROUGH<br>HOUSE FLY	LAGOON<br>TROUGH<br>STABLE FLY	LAGOON<br>HOUSE FLY<br>STABLE FLY	TROUGH<br>HOUSE FLY<br>STABLE FLY
5	5	6	5	6	5	3	5	4	3
Brettanomyces bruxellensis	Brettanomyces bruxellensis	Brettanomyces bruxellensis	Brettanomyces bruxellensis	Brettanomyces bruxellensis	Brettanomyces bruxellensis	Brettanomyces bruxellensis	Brettanomyces bruxellensis	Brettanomyces bruxellensis	Brettanomyces bruxellensis
Cantharellus sp	Diutina catenulata	Cantharellus sp	Morchella eximia	Cantharellus sp	Diutina catenulata	Oidium heveae	Cantharellus sp	Diutina catenulata	Oidium heveae
Oidium heveae	Geotrichum candidum	Diutina catenulata	Oidium heveae	Epichloe sylvatica	Geotrichum candidum	Puccinia arachidis	Clavaria fumosa	Geotrichum candidum	Pichia kudriavzevii
Onygenales sp	Oidium heveae	Geotrichum candidum	Pichia kudriavzevii	Oidium heveae	Oidium heveae		Oidium heveae	Oidium heveae	
Puccinia arachidis	Puccinia arachidis	Oidium heveae	Puccinia arachidis	Onygenales sp	Pichia kudriavzevii		Onygenales sp		
		Onygenales sp		Pichia kudriavzevii					

4-way

MANURE<br>LAGOON<br>TROUGH<br>HOUSE FLY	MANURE<br>LAGOON<br>TROUGH<br>STABLE FLY	MANURE<br>LAGOON<br>HOUSE FLY<br>STABLE FLY	MANURE<br>TROUGH<br>HOUSE FLY<br>STABLE FLY	LAGOON<br>TROUGH<br>HOUSE FLY<br>STABLE FLY
3	4	4	3	2
Brettanomyces bruxellensis	Brettanomyces bruxellensis	Brettanomyces bruxellensis	Brettanomyces bruxellensis	Brettanomyces bruxellensis
Oidium heveae	Cantharellus sp	Diutina catenulata	Oidium heveae	Oidium heveae
Puccinia arachidis	Oidium heveae	Geotrichum candidum	Pichia kudriavzevii	
	Onygenales sp	Oidium heveae		

5-way

MANURE<br>LAGOON<br>TROUGH<br>HOUSE FLY<br>STABLE FLY
2
Brettanomyces bruxellensis
Oidium heveae
